# Obstructive Sleep Apnea and Pulmonary Hypertension: A Review of Literature

**DOI:** 10.7759/cureus.14575

**Published:** 2021-04-20

**Authors:** Farhan A Shah, Shaidy Moronta, Michalla Braford, Nelson Greene

**Affiliations:** 1 Internal Medicine, Lewis Gale Medical Center, Salem, USA; 2 Internal Medicine, Edward Via College of Osteopathic Medicine, Salem, USA; 3 Pulmonary and Critical Care, Lewis Gale Medical Center, Salem, USA

**Keywords:** pulmonary hypertenion, obstructive sleep apnoea

## Abstract

Obstructive sleep apnea (OSA) is a disease process involving recurrent pharyngeal collapse during sleep, resulting in apneic episodes. Clinically, symptoms can include snoring, sudden awakening with a choking-like sensation, excessive somnolence, non-restorative sleep, difficulty in starting or maintaining sleep, and fatigue. It results in impaired gas exchange, subsequently causing various cardiovascular, metabolic, and neurocognitive pathologies. Historically, OSA has been underdiagnosed and undertreated, especially in women.

OSA is associated with WHO (World Health Organization) class III pulmonary hypertension (PH) or PH due to lung disease. PH is a concerning complication of OSA and thought to occur in roughly 20% of individuals with OSA. The pathogenesis of PH in OSA can include pulmonary artery vasoconstriction and remodeling. Patients suffering from OSA who develop PH tend to have worse cardiovascular and pulmonary changes. We present a thorough review of the literature examining the interplay between OSA and PH.

## Introduction and background

Obstructive sleep apnea (OSA) is characterized by recurrent pharyngeal collapse during sleep [1]. Complete collapse of the pharynx causes apnea, whereas partial collapse causes hypopnea [1]. OSA leads to abnormalities with gas exchange, resulting in oxygen desaturation, hypercapnia, and sleep disturbances, which contribute to the subsequent cardiovascular, metabolic, and neurocognitive consequences [1]. OSA is a very prevalent comorbidity found in many patients and is often underdiagnosed and undertreated. Many patients remain unaware of their underlying condition and as such are unable to be treated for it. Other barriers towards seeking treatment for OSA include the stigma related to snoring, and access to polysomnography (especially in remote communities and in the developing world) [2]. Primary care physicians may not pursue early diagnosis of OSA, especially if patients do not endorse subjective sleepiness and the classic characteristic of a high body mass index (BMI) [2]. This perception of the characteristic of the typical patient with OSA remains despite evidence on the contrary. For example, up to 50% of people with OSA are not obese and 25% of people with moderate OSA do not present with any form of sleepiness [2].

Patients who are particularly at risk of developing OSA include those who present with increased daytime sleepiness and as a result can be drowsy driving an automobile or may experience work-related accidents due to fatigue, cognitive difficulties related to fatigue, hypertension, nasopharyngeal narrowing, and pulmonary hypertension (PH). Patients suffering from OSA often report snoring, episodes of apneas, sudden awakening with a choking-like sensation, excessive somnolence, non-restorative sleep, difficulty in starting or maintaining sleep, fatigue, nocturia, memory loss, and a morning headache [1,2]. Chronic, loud snoring, increased obesity (especially nuchal obesity), and a neck size of >17 inches in males and >16 inches in females are associated with a higher risk of OSA [3].

OSA can develop as a result of several factors including decrease in the forces of the pharyngeal dilators, the negative inspiratory pressure generated by the diaphragm, and abnormalities within the upper airway anatomy, with common sites of obstruction located in the pharynx [3]. Airway failure can occur when individuals with OSA sleep on their back and the base of the tongue adheres to both the posterior pharyngeal wall and the soft palate [3]. An enlarged soft palate, a large tongue, an edematous uvula, redundant pharyngeal mucosal, and large tonsils are the most common reasons for snoring and OSA [3].

OSA is associated with WHO (World Health Organization) class III PH or PH due to lung disease [4]. PH is a concerning complication of OSA and thought to occur in roughly 20% of individuals with OSA, but the range is anywhere from 17% to 70% in the literature [5,6]. There are several factors that increase the risk of both PH and OSA, such as older age, obesity, left-sided heart disease, parenchymal lung disease, and nocturnal desaturation. The level of the pulmonary artery pressure correlates with the severity of OSA [5,7-10].

## Review

Epidemiology 

A major study examining the prevalence of OSA was the 1993 Wisconsin Sleep Cohort Study, which found that OSA is prevalent in approximately 4% of men and 2% of women among the middle-aged population and increases with age [1]. The prevalence is thought to range from 28% to 72% in elderly men and 20% to54% in elderly women [7]. These numbers are rough estimates and thought to be falsely low given how often OSA is undiagnosed [2]. Men have been found to be approximately three times more likely to have OSA; however, they are nine times more likely to be referred for polysomnography, which suggests that the diagnosis of OSA may be underdiagnosed in women [11].

The prevalence of obesity in the United States is increasing at a rapid rate. In 2017-2018, an estimated 42.4% of U.S. adults were obese as defined as a BMI of ≥30 [12]. Obesity is a risk factor for PH independent of the presence of OSA and can further complicate the picture of OSA. When PH occurs secondary to OSA, the symptoms may not be as clearly evident due to obesity and lack of physical activity [5]. Further complicating the picture, OSA is an independent risk factor for health complications such as myocardial ischemia, systemic hypertension, congestive heart failure, arrhythmias, and sudden cardiac death [5]. Therefore, PH symptoms can be overlooked in lieu of cardiovascular complications that take precedence [5]. Individuals who are obese are also subject to having obesity hypoventilation syndrome (OHS). OHS patients have a prevalence 59% greater of PH in OSA than having OSA alone, which is likely attributed to increased hypercapnic effects [5]. When weight loss therapies are implemented, OSA severity is reduced [13].

Pathophysiology 

The pathogenesis of PH in OSA is related to intermittent hypoxia, hypercapnia, acidosis, sympathetic hyperactivity, negative pleural pressure, and endothelial dysfunction [7,14]. The end result is pulmonary artery vasoconstriction and remodeling [14].

The mechanism behind PH and hypoxia is thought to relate to the duration of time in a hypoxic state. Individuals with OSA experience cyclical episodes of oxygen desaturation that last from 10 to more than 40 seconds each hour followed by arousal and either partial or complete recovery of oxygen saturation [5]. The episodes of hypoxia and hypercapnia in OSA result in a series of pulmonary arterial vasoconstriction and remodeling [14]. While the exact mechanism is unknown, levels of reactive oxygen species (ROS), known for vasodilatory effects, are reduced in acute hypoxia states. This process results in higher pulmonary arterial pressures [5]. Settings of chronic hypoxia are thought to result in vascular remodeling. The etiology of this process is attributed to endothelial nitric oxide synthase (eNOS) inhibition, which results in decreased levels of nitric oxide (NO), a vasodilator.

Vasoactive mediators, such as endothelin-1 and NO, are at lower levels in OSA patients [8]. Endothelin-1 is a potent vasoconstrictor that is produced by the endothelium and is typically increased in conditions of chronic intermittent hypoxia, as shown in animal studies, by binding to increased amounts of ETA (endothelin A) receptors on the pulmonary artery. Animal studies revealed that rats exposed to chronic intermittent hypoxia also had damaged endothelium, leading to less endothelium-dependent vasodilation. Similar effects occur with NO in the setting of chronic intermittent hypoxia with suppression of eNOS and impaired vasodilation of the endothelium, mediated by NF-kB (nuclear factor kappa B) mechanisms [5].

Certain anti-inflammatory hormones, such as adiponectin, are decreased in obesity and OSA, a possible linkage between conditions of pulmonary artery hypertension in OSA patients. Adiponectin is responsible for vasodilation of the pulmonary vessels. When levels are decreased, resultant systemic hypertension occurs. Decreased adiponectin is also associated with impaired vasoreactivity, which can further impact pulmonary vessels [14].

Given the multifactorial etiology between obesity and OSA, it is not surprising that OHS would be associated with PH as well. OHS is thought to contribute to PH through a series of mechanisms including diurnal hypoxemia, hypercapnia, and acidosis [14]. The degree of obesity present is thought to alter thoracic pressure changes in the respiratory cycle attributed to upper airway resistance. The resultant endothelial dysfunction, arterial wall thickening, and fibrosis lead to pulmonary artery remodeling and pulmonary artery hypertension [14].

Diagnosis 

The diagnosis of OSA is primarily made based on polysomnographic evidence observed in a sleep laboratory [11]. Home polysomnography is a viable option for those unable to carry out an overnight laboratory study, albeit it is less accurate and should not be utilized in patients with cardiovascular, pulmonary, or neurologic comorbidities [11]. Although the American Academy of Sleep Medicine suggests that signs and symptoms of OSA should be assessed in every patient, the U.S. Preventive Services Task Force makes no recommendation for general screening [15]. Risk factors that may solicit screening in the primary setting include adults aged 40 to 70 years, commercial motor vehicle operators, positive family history, BMI > 35, postmenopausal women not on hormonal therapy, retrognathia, and as a preoperative assessment for bariatric surgery [15]. Initial assessment of OSA often includes screening questionnaires such as the STOP-Bang questionnaire and the Epworth Sleepiness Scale (ESS) [11]. Despite the widespread use of ESS in primary care and as a subjective endpoint in clinical research, its performance pales in comparison to STOP-BANG with a sensitivity of only 39% versus 87%, respectively [15]. ESS only takes into account sleepiness, a cardinal symptom of OSA, but one that is hardly universal in all patients regardless of the severity of disease, whereaa STOP-BANG includes several subjective and objective factors predictive of OSA, including observed apnea, neck circumference, age, and gender [11,15].

During polysomnography, patients are monitored for episodes of apnea and hypopnea. Apnea is defined as a complete obstruction of airflow for greater than 10 seconds [11]. Hypopnea, on the other hand, is defined as a partial obstruction of airflow measured by an oxygen desaturation of at least 3% or by arousals from sleep, each lasting greater than 10 seconds [11]. These apneic and hypopneic episodes are summed and divided by total hours of sleep to give the apnea-hypopnea index (AHI). Mild OSA has an AHI of 5-15 and is characterized by involuntary somnolence during activities that demand little attention, including watching television or reading. Moderate OSA has an AHI of 15-30 and involves involuntary sleepiness during activities that require some attention, including meetings or presentations. Severe OSA has an AHI of >30 and includes involuntary somnolence during activities that demand an increased attention such as talking or driving [3].

PH has several etiologies, including OSA (WHO group III) [16]. Although AHI does not predict who will develop PH, those that do develop PH tend to have worse cardiovascular and pulmonary changes if the AHI is within the moderate-to-severe range versus mild [11]. The American College of Chest Physicians (ACCP) recommends screening for sleep-disordered breathing in patients found to have pulmonary arterial hypertension and subsequent polysomnography if OSA is suspected as the etiology [6,17]. However, there exist no formal guidelines for the screening of PH in patients diagnosed with OSA. Screening for PH of any etiology generally involves 2D echocardiography with Doppler flow studies, which is also helpful in excluding cardiac disease that may be contributory to increased pulmonary pressure [16]. Evidence of PH can also be gleaned from electrocardiogram (ECG) strips demonstrating signs of right ventricular hypertrophy (right axis deviation, tall right precordial R waves) as persistent pulmonary arterial hypertension subsequently leads to cor pulmonale [16]. Suggestive ECG findings should prompt echocardiographic investigation in these patients. Nonetheless, the gold standard for diagnosis of PH is based on right heart catheterization with current thresholds defined as a pulmonary artery pressure greater than 25 mmHg at rest or greater than 30 mmHg with exertion [9,17]. Although these studies are invasive, they may be of more utility in obese patients versus echocardiography due to body habitus obscuring proper interpretation. Likewise, right cardiac catheterization is essential to exclude left heart causes of PH for which vasodilatory therapies such as NO, adenosine, epoprostenol, or nitroprusside would be contraindicated [17].

Other studies that should be performed on patients with OSA and/or PH include routine biochemical, hematologic, and immunologic tests, thyroid function tests, pulmonary function tests, chest imaging, arterial blood gases, and ventilation-perfusions scans to exclude any other potentially reversible causes of disease [5,17].

Treatment 

OSA has been treated by several different modalities over the years including CPAP (continuous positive airway pressure), surgical interventions, medical treatment, and exogenous devices. The aim is mostly directed at reducing the number of apneic and hypopneic episodes, decreasing nocturnal arousals, and improving arterial oxyhemoglobin saturation [18]. CPAP is described as the first line and the gold standard treatment for OSA [19]. When used appropriately, CPAP reduces daytime sleepiness, normalizes sleep architecture, and improves morbidity and mortality associated with OSA, which may include the deleterious effects of PH [19]. Optimal treatment is described as 4 to 6 hours per night; however, many patients find it difficult to adhere to this due to comfort, convenience, noise, claustrophobia, cost, and so on [19]. The rate of adherence has been persistently low over the last 20 years despite behavioral intervention and patient coaching [19].

CPAP has long been the treatment of choice for OSA as it maintains the airway patent during sleep; however, improvement in PH has not been clearly established [8]. Two studies explored the relationship between CPAP therapy and PH in patients without underlying pulmonary or cardiac disease and found that pulmonary artery pressure significantly decreased after four months of treatment [8,20]. Arias et al. utilized a placebo control group and more extensive exclusion criteria, whereas Sajkov et al. did not. Nevertheless, patient demographics and ultimate findings were similar between each study. CPAP therapy was found to help restore normal serum level of vasoactive mediators, impacting pulmonary vascular tone and vascular smooth muscle proliferation [8]. The effect on left ventricular afterload and diastolic relaxation may be the component that is immediately reversed after 12 weeks of therapy, as seen in these studies [8]. However, further evaluation of the reversibility of pulmonary structural changes after a more prolonged course of treatment is warranted, especially in OSA cases with concurrent PH.

Although CPAP therapy is the standard, compliance rates are very low (30-60%), which makes finding alternative treatments for OSA and thus PH imperative [18,19]. Atrial overdrive pacing (AOP) in which a pacemaker is set to 15 beats per minute above a patient’s baseline nocturnal heart rate was one such alternative considered since there was a reported reduction in breathing disorders after implantation [21]. The prospective study by Garrigue et al. demonstrated that AOP in patients who had received pacemakers for the treatment of symptomatic sinus bradycardia and who also had sleep apnea had a significant reduction in their hypopnea index (the total number of episodes of hypopnea divided by the number of hours of sleep), decreased arousal, and increased arterial oxyhemoglobin saturation [21]. These findings offer an alternative treatment option for patients who already have a pacemaker in place for bradycardia and also happen to suffer from continued sleep apnea after CPAP failure. This study included patients with both central sleep apnea and OSA and reported improvement in indices for both. In terms of central sleep apnea, AOP may decrease periodic variations in heart rate by decreasing vagal tone response to hypoxemia, hypercapnia, bradycardia, and decreased blood pressure, leading to an increase in cardiac output and decrease pulmonary congestion [8,21]. Less variation in a patient’s heart rate was associated with a reduced number of apneic episodes. A mechanism for improved indices in OSA was not detailed in this study.

Three randomized controlled trials utilized this same treatment modality but only included patients with OSA rather than central or mixed sleep apnea. In stark contrast to the study by Garrigue et al., none of the three studies demonstrated significant improvement in AHI, respiratory indices, or subjective sleep quality parameters [18,22,23]. In addition to excluding patients with other sleep apnea syndromes, Simantirakis et al. also excluded any patient with clinical left ventricular systolic dysfunction and postulated that patients with mild left ventricular ejection impairment may have shown benefits to AOP due to improved cardiac function and reduction in periodic breathing [18]. Patient age in this study was also younger than the others, with a mean of 60±11 years compared with mean ages of 69±9, 71±10, and 74±6.6 years, further representing a healthier cohort overall [18,21-23]. These patients were randomly assigned to standard CPAP versus AOP therapy for one month with crossover after the initial therapy [18]. Although AOP did not exert a significant effect on OSA, this study did demonstrate that CPAP had a profound effect on AHI, respiratory indices, and ESS, further corroborating that CPAP is the superior treatment when patient compliance is optimal. Pépin et al. and Sharafkhaneh et al. did include patients with left ventricular heart failure in their randomized study, with the latter adopting this as an inclusion criterion. Sharafkhaneh et al. showed that AOP had a mild effect on respiratory events in this particular subset but could not demonstrate any further benefit of AOP treatment in terms of patency or sleep function [22]. Overall, it seems that AOP is not a suitable alternative to CPAP in patients with obstructive type sleep apnea but may indeed offer benefit for those with central or mixed apneas. Furthermore, AOP will likely not show improvement in OSA patients with concomitant or resultant PH due to lack of effectiveness in otherwise healthy OSA patients, although this parameter has not been effectively studied.

Since OSA is largely due to nocturnal anatomic disturbance of airflow, several surgical interventions have been employed to address the source of obstruction, including uvulopalatopharyngoplasty (UPPP), tonsillectomy, tracheostomy, radiofrequency ablation of tissue, and advancement and modification of skeletal and soft tissue structures [24].

The most common surgical procedure used in the treatment of OSA is UPPP since it is less invasive and relatively well tolerated [25]. This procedure involves the removal of excess tissue of the oral and nasopharyngeal mucosa and submucosa, shortening of the uvula, and sparing of the palatoglossal and palatopharyngeal muscles [25]. In the study by Langin et al., the main goal was to determine if there was an effective enlargement of the upper airway as a result of UPPP and whether this would result in improved AHI [26]. Although the majority of the patients in the study were considered poor responders pursuant to inadequate improvement in AHI, those who did herald a good response had an increase in the cross-sectional area of the oropharynx after surgical intervention [26]. Those who responded poorly to UPPP tended to have an increased width of the soft palate, which may be contributory to less than optimal response.

 Another study conducted by Boot et al. served to observe the long-term response of this intervention (UPPP alone and UPPP plus tonsillectomy) over a period that ranged over approximately six years [25]. The results of this study showed significant improvement in snoring but varied responses to other sleep quality parameters such as excessive daytime sleepiness and oxygen desaturation. However, when combined with tonsillectomy, measurements of nocturnal desaturation showed improvement that may correlate with an even further increased cross-sectional area of the oropharynx. As stated previously, increased cross-sectional area is correlated with a better response; nevertheless, not all patients who received additional tonsillectomy in this study were considered to have a good response. Surgeries such as UPPP may initially be beneficial and improve quality of life versus CPAP therapy; however, recurrence of excessive daytime sleepiness was evident in this cohort after six months postoperatively and at long-term follow-up, which may indicate decreased benefit after an extended post-surgical period.

UPPP remains effective in only around 20% of patients, and identifying “good responders” pre-operatively has been difficult to discern thus far [25,26]. A study conducted by Lee et al. investigated whether there was a difference in UPPP outcomes based on patients’ pre-operative positional dependency [27]. Positional dependency is ascribed to an OSA patient when signs and symptoms are more pronounced in the supine position relative to lateral positioning (demonstrated by a difference in supine AHI > 2 times that of the lateral position) [27]. Therefore, positional independence is seen with more severe OSA, and based on this study, this was the population that benefitted the most from UPPP intervention. An improvement in AHI indices was seen in both position-dependent and position-independent groups; however, the change was more pronounced in the position-independent cohort in both the supine and lateral positions, with the majority of these patients actually becoming position-dependent after intervention [27]. Therefore, assessing positional dependency pre-operatively could serve as a beneficial selection tool to stratify those most likely to reap the benefits of UPPP and potentially other surgical treatments for OSA.

Besides UPPP, other surgical interventions included in this paper are radiofrequency ablation of obstructing tissue, and upper airway stimulation system implants. Radiofrequency ablation can be directed towards the tongue, turbinates, or soft palate, and usually does not require general anesthesia [24]. However, the drawback of this kind of intervention is that multiple sessions may be needed before improvements in AHI and subjective sleep efficiency parameters could be seen [24]. For example, Powell et al. used an electrode to deliver temperature-controlled radiofrequency to reduce the volumetric mass of palatal submucosa in 22 non-randomized patients with sleep-disordered breathing and found a significant improvement in esophageal pressures, sleep efficiency, snoring, and ESSs with an average of 3.6±1.2 treatment sessions [28]. In contrast, no significant improvement in OSA parameters was found in a randomized controlled trial conducted by Bäck et al [29]. Recommendation for radiofrequency ablation as an alternative warrants further investigation but may continue to serve as an adjunct to standard treatment.

While the previous surgical interventions discussed serve to permanently decrease the effective mass of tissue periodically blocking the oropharynx in OSA, upper airway stimulation system implants preserve the anatomic structure of the oropharynx and instead delivers an impulse to mechanically reduce obstruction. While OSA is traditionally defined by apnea and hypopnea caused by soft palate collapse, hypoactivation of the genioglossus muscle is also correlated with decreased patency of the airway as relaxation of the muscle causes the tongue to retract posteriorly and cause obstruction [30]. Strollo et al. investigated the effectiveness of hypoglossal nerve stimulation in correcting the periodic obstruction caused by tongue base displacement by way of an implantable electrode wrapped around the hypoglossal nerve [30]. Of the 126 patients who had undergone intervention, only 46 had a good response and were then randomized to treatment maintenance or treatment withdrawal. During this time, no significant difference between AHI was noted, leading investigators to conclude that improvement in overall AHI score was due to the year-long hypoglossal nerve stimulation rather than variability in AHI score [30]. This study was funded and partially designed by Inspire Medical Systems. Less invasive treatment modalities that also target tongue-based obstruction include mandibular advancement splints and tongue stabilizing devices [31]. Both modalities showed a significant reduction in AHI and subjective sleep parameters in a study by Deane et al., but they may have subsequent issues with compliance as seen in CPAP since it requires the user to use an intra-oral device during sleep.

A comparison of the various treatment modalities for treating underlying OSA can be seen in Table 1.

**Table 1:** Various treatment modalities for OSA AHI, apnea–hypopnea index; AI, apneic index; AOP, atrial overdrive pacing; BMI, body mass index; bpm, beats per minute; CPAP, continuous positive airway pressure; ESS, Epworth Sleepiness Scale; FOSQ, Functional Outcomes of Sleep Questionnaire; HTN, hypertension; LVEF, left ventricular ejection fraction; MAS, mandibular advancement splint; ODI, oxygen desaturation index; OSA, obstructive sleep apnea; PFT, pulmonary function test; Ppa, pulmonary artery pressure; PSG, polysomnography; RCT, randomized controlled trial; TSD, tongue-stabilizing device; UPPP, uvulopalatopharyngoplasty

Intervention	Study	Study type/year	Sample size	Patient demographics	OSA parameters used in study	Inclusion criteria	Exclusion criteria	Duration of treatment	Study design	Findings
CPAP	Sajkov et al. [20]	Prospective/2000	22 (20 completed study)	21 males, mean age 49.9±2.5 years, mean AHI 48.6±5.2/h	Hypopnea: ≥50% decrease from baseline in abdominal and thoracic excursion. Apnea: cessation of airflow for ≥10 sec. AHI, SaO_2_	Moderate-to-severe OSA	Lung or cardiac disease, systemic HTN	4 months	Treatment naïve OSA patients were started on CPAP therapy and several parameters measured.	Ppa significantly decreased after 4 months of CPAP therapy. Ppa 16.8±1.2 mm Hg before CPAP vs. 13.9±0.6 mm Hg after CPAP, p<0.05. Hypoxic vascular reactivity significantly reduced after CPAP (total pulmonary vascular resistance before treatment 231.1±19.6 vs. 186.4±12.3 dyn·s·cm^−5^ after treatment, p<0.05).
	Arias et al. [8]	RCT/crossover/2006	23	96% male, mean age 51±13 years, mean BMI 30.9±4, mean AHI 44.1±29.3/h	Hypopnea as above and associated with a fall in SaO_2_>4% of baseline; apnea as above; sleep time with SaO_2_<90% on nocturnal oximetry	AHI≥10/h; ESS ≥10 points; no previous treatment for OSA	Obstructive or restrictive lung disease on PFT, connective-tissue or chronic thrombo-embolic diseases, current cardioactive drugs, cardiac rhythm disturbances, known HTN, LVEF<50%, ischemic or valvular heart disease, cardiomyopathy, pericardial disease, stroke, BMI>40 kg/m^2^, daytime hypoxemia or hypercapnia, history of cocaine or appetite-suppressant drug use	12 weeks	Patients randomized to receive 3 months of sham CPAP or CPAP, then crossover for another 3 months.	The level of Ppa maintained a direct relationship with both the severity of OSA and the presence of LV diastolic dysfunction. Middle-aged OSA patients had higher baseline pulmonary artery systolic pressure than control (29.8±8.8 vs. 23.4±4.1 mmHg, p=0.036). Effective CPAP treatment for 12 weeks led to a significant reduction in pulmonary systolic pressures compared to sham treatment (28.9±8.6 to 24.0±5.8 mmHg, p<0.0001).
AOP	Garrigue et al. [21]	Prospective/2002	15	11 males, 4 females, mean age 69±9 years, LVEF 54±11, central sleep apnea or OSA (>50% had central sleep apnea)	Apnea index≥5; AHI≥15; apnea as above. Hypopnea: ≥50% decrease in oronasal air flow with a 4% decrease in SaO_2_.	Patients who received permanent atrial-synchronous ventricular pacemakers for symptomatic sinus bradycardia.	No clinical evidence of heart failure, pacemaker dependent.	3 nights	3 PSG: 1 for baseline, 1 night of basic ventricular rate of the pacemaker at 40 bpm (not synchronized with atrial activity), 1 night of AOP (15 bpm above patient's baseline).	The hypopnea index was reduced from 9±4 in spontaneous rhythm to 3±3 with atrial overdrive pacing (p<0.001). Total sleep time was similar at baseline and during the pacing and no-pacing phases (321±49 minutes in spontaneous rhythm vs. 331±46 minutes with AOP, p=0.48).
	Simantirakis et al. [18]	RCT/crossover/2005	16	12 males, mean age 60±11 years, mean AHI 49±19/h	Based on PSG	Moderate-to-severe OSA and sleep-related bradyarrhythmia, >2 syncopal events in previous year, normal LV systolic function, dual-chamber pacemaker	Heart failure, LVEF<50%, central sleep apnea	24 hours and 1 month	Patients initially given atrial overdrive pacing (15 bpm above baseline nocturnal heart rate) or backup atrial pacing if HR<40 bpm + CPAP; crossover after one month.	During AOP, no significant changes were observed in any of the respiratory variables measured (arousal index, mean or lowest SaO_2_, total sleep time, or PaCO_2_). Change in AHI at one month with AOP was +0.2 (p=0.87). All variables improved after one month of CPAP (change in AHI decreased from 49.0 to 2.7, p<0.001). ESS also changed significantly in the CPAP period vs AOP (net change -10.2, p<0.001 vs net change +0.1, p=0.67).
	Pépin et al. [23]	RCT/crossover/2005	17 (15 completed study)	12 males, mean age 71±10 years, mean BMI 27±3, 67% of patients had AHI>30/h; 5 out of 15 had reduced LVEF (mean 64±13).	AHI>15/h but <30/h represents moderate OSA, AHI>30/h represents severe OSA; hypopnea: ≥50% decrease from baseline in abdominal and thoracic excursion or >30% reduction in nasal pressure signal associated with desaturation of >3% and/or a microarousal.	Patients who had received permanent atrial-synchronous ventricular pacemakers for symptomatic bradyarrhythmias, undiagnosed central sleep apnea or OSA.	-	1 month	AOP (15 bpm above baseline nocturnal heart rate) vs spontaneous rhythm.	Nocturnal spontaneous rhythm was 59±7 bpm at baseline vs 75±10 bpm with AOP (p<0.001). AHI for spontaneous rhythm was 46±29/h vs 50±24/h with AOP (p>0.05). Overdrive pacing changed none of the respiratory indices, sleep fragmentation, or sleep structure parameters.
	Sharafkhaneh et al. [22]	RCT / 2007	15	Mean age 74±6.6 yrs; LVEF 38±14.4, mean AHI 34.8±15.5/h	AHI>15/h	Patients with OSA and systolic heart failure	-	3 nights	1 night of positive airway pressure titration, 1 night of AOP, and 1 night of pacing set at 40-50 bpm in randomized order.	Average heart rate was significantly higher on AOP nights compared to paced nights (p<0.0005). AI was significantly lower on AOP nights (18±16.6 vs. 24±18.9, p<0.05). However, AHI and minimum and average O_2_ saturations did not differ significantly between AOP and backup paced nights. Overdrive pacing exerts a mild effect on respiratory events in some heart failure patients with OSA but not effective for improving airway patency and sleep-related respiratory function.
Surgical	Langin et al. [26]	Prospective/1998	20	Mean age 45±2 years; mean BMI 26±4	Good responders: AHI<10 postsurgery or reduction in AHI>50% of the initial AHI.	-	-	10±10 months after surgery	PSG before and 10 months after UPPP; pharyngeal CT imaging studies to measure cross-sectional area of the hard palate.	Based on PSG criteria, 35% were good responders and 65% were non-responders. BMI and AHI were unchanged after UPPP (14±13 vs 18±16/h). Imaging showed a decrease in length (40±6 vs 29±5 mm, p≥0.0006) and increase in width of the soft palate (11.5±2.7 vs 13.6±3.5 mm, p≤0.006). Changes in cross-sectional area were significant only in patients who responded favorably to UPPP (7 vs 13 nonresponders).
	Powell et al. [28]	Nonrandomized prospective study/1998	22	18 males, mean age 45.3±9.1 years	-	Respiratory disturbance index≤15, oxygen saturation≥85%, and a complaint of daytime sleepiness	-	Endpoint of study = when individual snoring scores were reduced (mean of 3.6±1.2 treatments per patient).	Evaluate response after radiofrequency treatment to the palate via PSG, radiographic imaging, infrared thermography, questionnaires, visual analog scales.	Esophageal pressure and mean sleep efficiency index were improved (p=0.031 and p=0.002, respectively). Imaging showed mean shrinkage of 5.5±3.7 mm (p≤0.0001). Snoring decreased by 77% (8.3±1.8 to 1.9±1.7, p=0.0001) and ESS also showed improvement (8.5±4.4 to 5.2±3.3, p=0.0001).
	Boot et al. [25]	Prospective/2000	58	51 males, 7 females, mean age 49 years, mean BMI 29.6, 19 underwent UPPP alone, 39 had UPPP+tonsillectomy	Apnea>10 seconds leading to >5 desaturations/hour of sleep or >3% reduction in baseline oxygen saturation	Confirmed OSA	CPAP use, abnormal facial anatomy, concomitant disease	11-74 months	Compare long-term response vs response at 6 months using snoring and daytime sleepiness questionnaires and desaturation parameters.	Response to UPPP in this cohort showed long-term improvement in snoring in 63%, no change in snoring in 23%, and deterioration in 14% (p<0.00001). Change in excessive daytime sleepiness insignificantly improved in 38%, heralded no change in 27%, and deteriorated in 35% (p=0.80). Nocturnal ODI decreased with UPPP in combination with tonsillectomy, compared with a slight increase with UPPP alone (p=0.008).
	Lee et al. [27]	Retrospective/2011	96 (74 completed study)	72 males, 2 females, mean age 47 years, mean overall pre-operative AHI in position-independent group was 50.0 vs 30.9 in position-dependent group (p<0.001). Average lateral AHI was 46.3 in position-independent group vs 9.7 in position-dependent group (p<0.001). No significant difference between supine AHI for either group.	Positional dependence = if AHI in supine was twice that of AHI in lateral position.	Patients who underwent UPPP from June 1, 2004, through July 31, 2008.	If spent <5% of total sleep time in either supine or lateral position.	6-12 months	Compared UPPP results between 22 position-independent patients vs. 52 position-dependent patients. Surgical success = reduction in AHI≥50% and a post-operative AHI of ≤20.	Overall AHI declined from 50.0 to 35.4 in position-independent patients (p=0.045) and from 30.9 to 22.9 in position-dependent patients (p=0.002). AHI in the lateral position was markedly reduced after UPPP in patients who were position-independent from 50.0 to 35.4 (p=0.02). 64% of patients with position-independent OSA acquired positional dependency after UPPP.
	Strollo et al. [30]	Prospective/RCT/2014	126 (46 were then assigned to RCT after 12 months)	83% male, mean age 54.5 years, mean BMI 28.4, 17% had previously undergone UPPP, mean AHI prior to implant was 32.0, mean ODI 28.9, mean FOSQ 14.3, mean ESS 11.6	AHI≥15 indicating moderate-to-severe apnea, ODI drops by ≥4% from baseline, ESS, FOSQ, percentage of sleep time with SaO_2_<90%	Moderate-to-severe OSA with difficulty accepting or adhering to CPAP.	AHI<20 or >50/h, if central or mixed sleep-disordered breathing events accounted for >25% of all apnea and hypopnea episodes, supine AHI<10/h, pronounced anatomic abnormalities, BMI>32, neuromuscular disease, hypoglossal-nerve palsy, severe restrictive or obstructive pulmonary disease, moderate-to-severe pulmonary arterial HTN, severe valvular heart disease, NYHA class III or IV, recent myocardial infarction or severe cardiac arrhythmias (within the past 6 months), persistent uncontrolled HTN despite medication use, active psychiatric disease, and coexisting nonrespiratory sleep disorders	12 months + 7 days	Eligible patients were implanted with upper-airway stimulation system and had outcomes and adverse events recorded at 2, 3, 6, 9, and 12 months. Those who were responsive to therapy after 12 months were then randomly assigned to a therapy-maintenance group or a therapy-withdrawal group for 7 days.	Median AHI decreased 68% from 29.3/h to 9/h (p<0.001). Median ODI decreased 70% from 25.4/h to 7.4/h (p<0.001). FOSQ and ESS showed improved quality of life and reduced effects of sleep apnea. In the randomized phase, the mean AHI score did not differ significantly from the 12-month score in the nonrandomized phase among the 23 participants in the therapy-maintenance group (8.9 and 7.2 events per hour, respectively); the AHI score was significantly higher (indicating more severe apnea) among the 23 participants in the therapy-withdrawal group (25.8 vs. 7.6 events per hour, p<0.001).
Mandibular device	Deane et al. [31]	RCT crossover/2009	27 (22 completed study)	20 male, 7 female, mean age 49.4±11.0 years, BMI 29.3±5.6, baseline AHI 27.0±17.2, baseline minimum SaO2% 84.3±6.5	OSA symptoms: snoring, fragmented sleep, witnessed apneas, daytime sleepiness; AHI: cessation of airflow>10 seconds with oxygen desaturation>3% and/or associated arousal. Hypopnea: reduction in airflow >50% of baseline tidal volume for >10 seconds. Mild OSA: AHI 5-15/h, Moderate OSA: 15-30/h, Severe OSA: AHI >30/h	>20 years old, ≥ 2 symptoms of OSA, evidence of OSA on PSG	Regular use of sedative medications, previous failure of an oral device for OSA treatment, exaggerated gag reflex, edentulous patients, <10 teeth per jaw, periodontal disease	8 weeks (4 weeks with each device in random order + 1 week washout period)	Compare efficacy of MAS and TSD in treating OSA. Complete response: resolution of symptoms and reduction of AHI<5/h. Partial response: improved symptoms and >50% reduction in AHI but still remained >5/h. Failure: ongoing clinical symptoms and/or reduction in AHI<50%.	AHI was reduced with MAS (11.68±8.94, p=0.000) and TSD (13.15±10.77, p=0.002) compared with baseline (26.96±17.17). ESS score decreased with both MAS and TSD interventions (p<0.001 and p=0.002, respectively). 68% achieved complete or partial response with MAS compared with 45% with TSD. Compliance was better with MAS with 91% of patients preferring this treatment over TSD.

## Conclusions

Novel treatments for OSA are actively being investigated in an effort to deliver an effective option that both reduces the deleterious effects of this disease and provides a sustainable method to increase patient satisfaction and adherence. Years of research have culminated in the institution of CPAP as the first-line treatment for sleep apnea and has led to a number of studies investigating its concurrent therapeutic effects on PH. Studies of this nature are severely lacking for alternative therapeutic measures in the treatment of OSA. The challenge remains in finding and establishing a viable alternative to CPAP that is effective in a sizable proportion of those treated and evaluating its effects on PH. Notably, pulmonary disease has served as exclusionary criteria for many studies, but moving forward we believe that it would be beneficial to include a cohort of OSA patients with PH in the investigation of alternative treatment options for OSA. Correlations may even be found that stratifies “good” and “poor” responders to novel treatments by initial presence or absence of PH. PH remains a devastating consequence of the constellation of disease pathologies originating from OSA. Since many patients suffering from OSA are underdiagnosed and undertreated, so too may be numerous concurrent cases of PH. Clinicians need to continue to consider the strong association between OSA and underlying PH when diagnosing and treating patients with either disease processes.
